# Generalizability concerns in the vitamin D and sarcopenia link

**DOI:** 10.1016/j.jnha.2025.100741

**Published:** 2025-11-29

**Authors:** Yi Zou, Qi Yang, Xiping Wang, Xiaokun Tang, Mei Yang

**Affiliations:** Anyue County People’s Hospital, Ziyang 642350, Sichuan, PR China

To the Editor,

We read with interest the recent article by Song et al. published in The Journal of Nutrition, Health and Aging, which investigated the sex-specific associations between serum 25-hydroxyvitamin D and incident sarcopenia in older Korean adults [1]. The authors are to be commended for their longitudinal design and thorough evaluation of muscle parameters. Nevertheless, several methodological aspects warrant further consideration to better contextualize the findings for clinical practice.

A primary concern is the generalizability of the results to older adults with significant multimorbidity. The study excluded individuals with conditions such as renal disease, cancer, or malnutrition—subgroups frequently encountered in geriatric care that may exhibit distinct vitamin D metabolism and sarcopenia trajectories. For example, Souberbielle et al. highlighted that vitamin D's effect on musculoskeletal outcomes is often attenuated in patients with chronic kidney disease due to impaired activation [2]. The exclusion of such clinically relevant populations may limit the translation of these findings into real-world practice. Future studies incorporating stratified analyses by comorbidity burden would be valuable.

Additionally, the assessment of muscle function relied predominantly on grip strength and gait speed. While these are valid measures, they may not fully capture functional limitations related to balance or endurance, which are critical in geriatric assessment. The SPPB combines standing-balance, short-walk (gait speed) and repeated chair-stand tests; while it does not directly measure maximal cardiopulmonary endurance, its gait-speed and chair-stand components partly reflect functional endurance and lower-limb power, and therefore provide a broader assessment than grip strength alone [3,4].

Finally, although the authors adjusted for several confounders, residual confounding from unmeasured lifestyle factors—such as dietary protein intake and structured physical activity—cannot be ruled out. Previous studies have emphasized that nutritional status, physical activity, and functional capacity are key determinants of muscle health in older adults and are often heterogeneous across populations [4,5]. Accounting for these lifestyle-related factors in future analyses would therefore help clarify the independent role of vitamin D in the development of sarcopenia. In summary, Song and colleagues provide meaningful insights into the sex-specific role of vitamin D in sarcopenia. Addressing these aspects in subsequent studies could further strengthen the clinical applicability of their conclusions.

## Ethics approval and consent to participate

Not applicable.

## Consent for publication

All authors gave their consent for publication.

## Funding

There is no funding.

## Availability of data and materials

Not applicable.

## Declaration of competing interest

The authors declare that they have no known competing financial interests or personal relationships that could have appeared to influence the work reported in this paper.
